# An automated workflow for quantifying RNA transcripts in individual cells in large data-sets

**DOI:** 10.1016/j.mex.2017.08.002

**Published:** 2017-09-01

**Authors:** Matthew C. Pharris, Tzu-Ching Wu, Xinping Chen, Xu Wang, David M. Umulis, Vikki M. Weake, Tamara L. Kinzer-Ursem

**Affiliations:** a Weldon School of Biomedical Engineering, Purdue University, United States; b Department of Biochemistry, Purdue University, United States; c Purdue University Center for Cancer Research, Purdue University, United States

**Keywords:** Cell-by-cell relative integrated transcript (CCRIT) quantification, smFISH, RNAscope, mRNA transcription, Machine learning

## Abstract

Advanced molecular probing techniques such as single molecule fluorescence in situ hybridization (smFISH) or RNAscope can be used to assess the quantity and spatial location of mRNA transcripts within cells. Quantifying mRNA expression in large image sets usually involves automated counting of fluorescent spots. Though conventional spot counting algorithms may suffice, they often lack high-throughput capacity and accuracy in cases of crowded signal or excessive noise. Automatic identification of cells and processing of many images is still a challenge. We have developed a method to perform automatic cell boundary identification while providing quantitative data about mRNA transcript levels across many images. Comparisons of mRNA transcript levels identified by the method highly correlate to qPCR measurements of mRNA expression in *Drosophila* genotypes with different levels of Rhodopsin 1 transcript. We also introduce a graphical user interface to facilitate analysis of large data sets. We expect these methods to translate to model systems where automated image processing can be harnessed to obtain single-cell data.

The described method:

• Provides relative intensity measurements that scale directly with the number of labeled transcript probes within individual cells.
• Allows quantitative assessment of single molecule data from images with crowded signal and moderate signal to noise ratios.

## Graphical abstract

**Figure:**
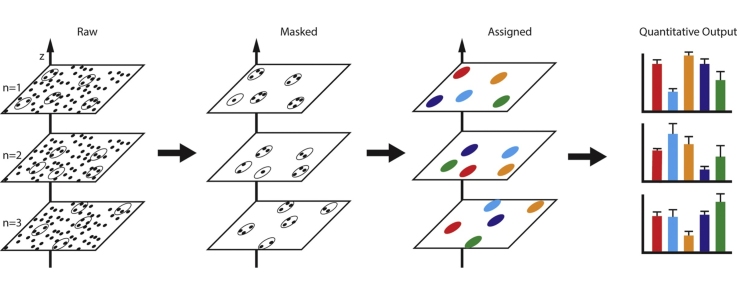

A noisy image of fluorescently-labeled mRNA transcripts can be analyzed by Cell-by-Cell Relative Integrated Transcript (CCRIT) Quantification to automatically identify cells and cell clusters and quantify each cell’s mRNA expression level.

## Method details

Precise descriptions of the spatial and temporal expression of genes via mRNA probing methods have become a valuable tool in developmental and disease biology. Understanding gene regulation events relevant to embryonic development, aging and disease pathology at the sub-cellular level requires more detailed analysis of transcriptome regulation than can be obtained by simple (e.g. biotin-labeled) *in situ* hybridization techniques [1], [2], [3]. Indeed, typical fluorescent probes for mRNA transcripts may exhibit poor sensitivity and non-specific binding [4]. Advances in probe technologies such as single molecule fluorescent *in situ* hybridization (smFISH) and RNAscope improve on conventional probing methods by requiring many molecular probes to bind a single target, improving selectivity and signal-to-noise ratio of the fluorescent measurements [5], [6], [7]. smFISH probes are small and specific for a range of sites along target mRNA transcripts, allowing many probes to simultaneously bind [4]. While binding of many smFISH probes increases the fluorescent signal from each target transcript, non-specific binding of these probes may lead to considerable background noise distributed uniformly within cells. In contrast to smFISH, RNAscope probes target mRNA transcripts with a sequence of pre-amplifier and amplifier molecules to which many probes can simultaneously bind [6].

smFISH and RNAscope are distinct methods, and both can provide individual mRNA counts when analyzed with image processing software. mRNA transcripts appear in images as small clusters of pixels with intensities greater than background noise. The transcripts appear as unresolved clusters because of the imaging system’s impulse response, characterized by a pointspread function. Quantification of the number and spatial distribution of individual mRNA transcripts can be obtained using confocal microscopy imaging in combination with spot counting algorithms [8], [9], [10], [11], [12], [13], [14]. Typically, transcripts are quantified by: (1) Image masking/segmentation, (2) Laplacian of Gaussian (LoG) filtering, (3) intensity thresholding, and (4) spot counting [4]. Widely used tools for analysis include FISH-QUANT, which is freely available software packages [8]. These packages greatly streamline image analysis and mRNA spot identification. In analysis, image masking is optional but often desirable to reduce background noise by imaging DAPI or similar stain in a separate channel, thereby visualizing nuclei boundaries. Nuclei boundaries can be readily identified using the Moore-Neighbor tracing algorithm modified by Jacob’s stopping criteria implemented in Matlab using the bwboundaries function [15]. Additional user input is needed when identifying the appropriate filter and pixel intensity threshold parameters. The advantage is that individual spot count data are obtained. But, quantification of mRNA transcript levels can be challenging and time consuming when analyzing large sets of data with crowded signal or with low signal to noise ratios. Automated quantification is attractive, because confocal microscopy often leads to large data sets. Therefore there is a need to develop methods to automatically identify individual cells and provide quantitative mRNA transcript level data.

Developing an automated workflow to count transcripts within a multi-stack of confocal images is essential to rapidly analyze large data sets. Problematically, the signal-noise ratio from confocal microscopy can decrease with imaging depth, often requiring that during image processing, filtering parameters be adjusted for each successive slice in a multi-stack. To account for this, automated workflows must intelligently choose and iterate the filtering parameters. Such parameters include masking tolerance, Gaussian scaling factor, requisite pixel connectivity for spot identification, and intensity threshold selection criteria. Especially in noisy data, subtle parameter shifts can drastically influence the reported transcript spot count. One solution for mitigating large shifts in reported spot counts between consecutive z-slices is to design a processing algorithm to iteratively re-assess images using an expanded parameter space. However, iterative parameter optimization is not ideal as it can add greatly to computing time and analytical uncertainty.

These challenges apply to conventional, Laplacian of Gaussian (LoG) filtering programs built in-house and currently available software packages that involve LoG filtering such as FISH-QUANT [8]. To address these challenges we have developed a new algorithm that uses automatic segmentation to identify and assign cell regions and reports an integrated intensity metric for comparing mRNA expression in individual cells [10]. Total integrated intensity should directly scale with the number of transcripts because single molecule imaging methods rely on a finite number of labeled probes [6], [11]. We present data that supports using integrated intensity from the single molecule probes as a viable quantitative output, even when individual transcripts are not resolvable. Herein, we refer to it as CCRIT (Cell-by-Cell Relative Integrated Transcript quantification) and document the development and validation of the approach.

### Automated conventional spot counting method

**Fig. 1:**
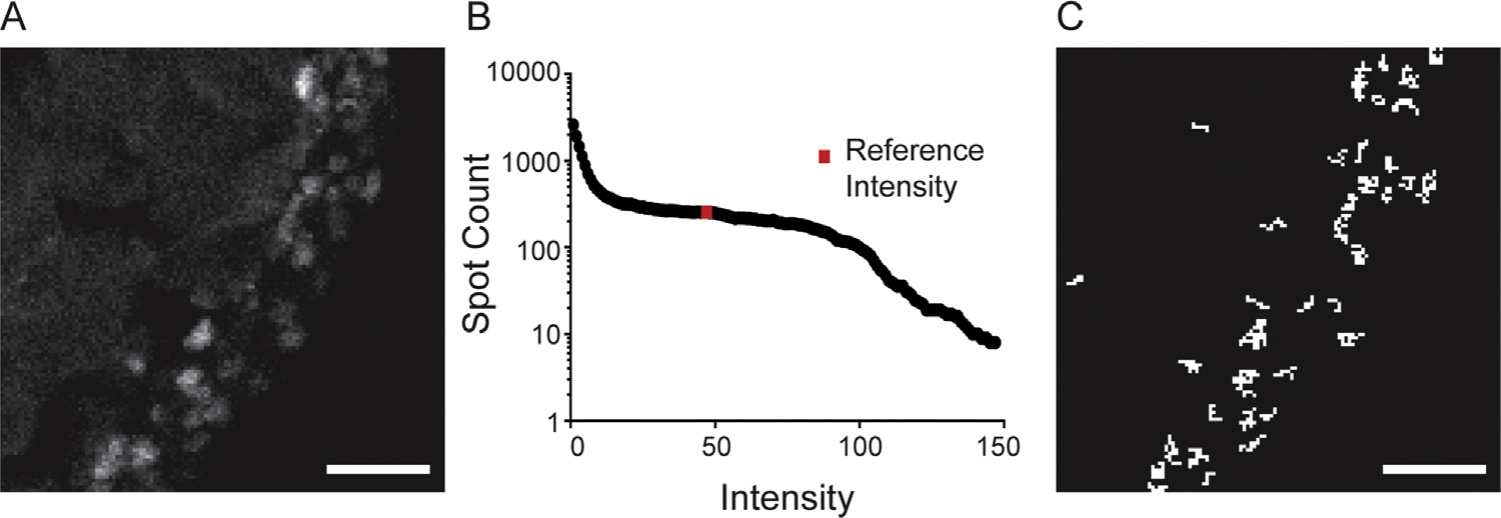
Conventional Spot Counting Analysis using in-house software on Matlab. A) Window of original image of zebrafish embryo cross-section labeled for *bmp2b* by RNAscope, obtained by confocal microscopy. B) Spot-Intensity distribution following LoG filtering. Note the non-zero plateau domain and log-scale on the vertical axis. Red point denotes the automatically-selected reference intensity. C) Processed (binary) image, thresholded at the reference intensity. Counted spots are shown in white. Scale bars denote 10 μm.

We built an in-house algorithm in Matlab to perform automated spot counting according to the conventional image processing steps. To validate the method, the image processing steps of the algorithm were applied to cross-sectional images of zebrafish embryos labeled with RNAscope probes for the *bmp2b* gene (Fig. 1A). The 2-dimensional Gaussian filter convolved the data with a Gaussian function to smooth the data while the Laplacian function served to exaggerate rapid shifts in signal intensity, making greater pixel intensity regions even more distinct from the background. Intensity thresholding served to differentiate background signal from regions of high pixel intensity. This threshold was determined by calculating the distribution of spots identified in a LoG-filtered image as a function of pixel intensity (Fig. 1B). The distribution should exhibit a non-zero plateau region denoting pixel intensities that are most resilient to noise. A threshold from within this plateau domain was automatically selected using the minimum discrete derivative and used for the final reported spot (putative transcript) count (Fig. 1C).

Ideally this automated algorithm would accurately identify mRNA transcripts for data acquired using either smFISH or RNAscope labeling methods. While we first implemented this algorithm to analyze images of cross sections of zebrafish embryos labeled by RNAscope for the *bmp2b* gene, we found that the results in Fig. 1 represent a best-case result. We observed that for images exhibiting non-noisy signal, such as these RNAscope images, this automated LoG spot counting algorithm is effective. In particular, the non-negative plateau region, used for selection of the reference intensity (indicated in red in Fig. 1B), was robust for many specimens and across a considerable range of filtering parameters.

### Conventional spot counting applied to crowded data

**Fig. 2:**
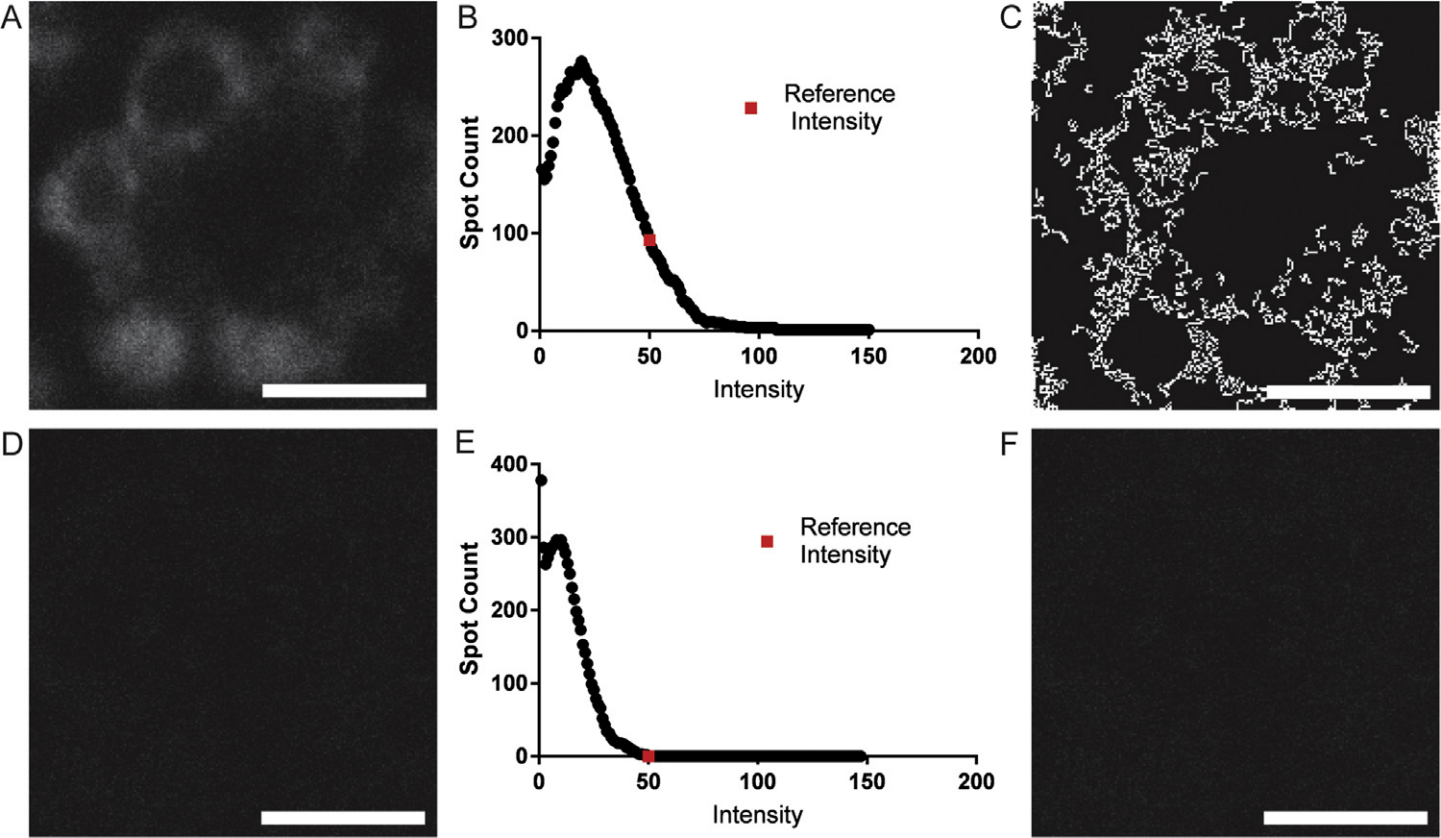
Analysis of *Drosophila* images by Conventional Spot Counting. Top row: (A) Original image of wild type (*w^1118^*) Drosophila photoreceptor neurons. (B) LoG-filtered spot-intensity distribution. (C) Processed (binary) image, thresholded at the reference intensity. Counted spots are shown in white. Bottom row: (D–F) *ninaE^0117^* homozygous mutant. (D) Processed image of *ninaE^0117^* photoreceptor neurons. (E) Spot intensity distribution and (F) processed (binary) image, thresholded at the reference intensity. Lack of white spots indicate no Rh1 transcripts are found as expected in this homozygous knockout. Scale bars denote 2 μm.

To further extend the utility of our automated spot counting algorithm, we applied the method to identify mRNA transcript expression for Rhodopsin 1 (Rh1) (*ninaE* gene) in the adult *Drosophila melanogaster* eye imaged using smFISH probes. Each ommatidium in the compound eye of *Drosophila* contains a mixture of eight different photoreceptor neurons together with several accessory cells [16]. Rh1 is highly expressed in a subset of photoreceptor cells (R cells, R1–R6) in wild type flies (*w^1118^)* [17], [18], [19]. The *ninaE*^*o117*^ allele of Rh1 lacks all detectable transcript, providing us with an appropriate negative control in whole-mount preparations of adult retina [18]. To examine Rh1 transcript levels in individual photoreceptor cells in an intact compound eye, smFISH was performed on whole-mount retina preparations from adult flies with probes designed against Rh1 mRNA; images were obtained by confocal microscopy using a LSM710 (Zeiss) confocal microscope with a 63× lens and 4× zoom. The pinhole size was set to 90 μm with a numerical aperture of 1.4. For each eye, z-stack images of 0.65 μm slices for 20 μm total depth were collected at pixel resolutions of 1024 × 1024 (see Supplemental material for more details of the experimental methods and imaging parameters). First, we examined white-eyed flies that are otherwise wild-type for Rhodopsin 1 (*w^1118^*) with *Rh1* smFISH probes. As expected we observed fluorescently-labeled spots only in the appropriate photoreceptors R1-R6 and not in R7 and R8, nor in other regions of the brain (Fig. 2A). This expression pattern was consistent with the known expression of Rh1 and indicated that our smFISH probes are specific for this gene. To test if smFISH could be used to accurately quantify Rh1 transcript levels in this tissue, we next examined Rh1 transcript levels in homozygous mutant flies that lack detectable Rh1 transcription (*ninaE*^*o117*^*)* [17]. We did not visually observe any fluorescent spots in the homozygous *ninaE*^*oI17*^ mutant eyes stained with smFISH probes against *Rh1*, supporting the specificity of the probes for this gene (Fig. 2D).

We analyzed smFISH confocal microscopy images from each *Drosophila* genotype using the automated conventional spot counting method described in the previous section, at a consistent imaging depth for each specimen. Although our conventional method did not identify any mRNA spots for the homozygous *ninaE^o117^* mutant (Fig. 2F) as expected, analysis of the wild type specimen yields non-intuitive results (Fig. 2C). As seen in Fig. 2C, the algorithm failed to identify spots in locations where they clearly existed as seen by visual inspection of the original image (Fig. 2A).

### Comparison of our automated spot counting to FISH-QUANT

**Fig. 3:**
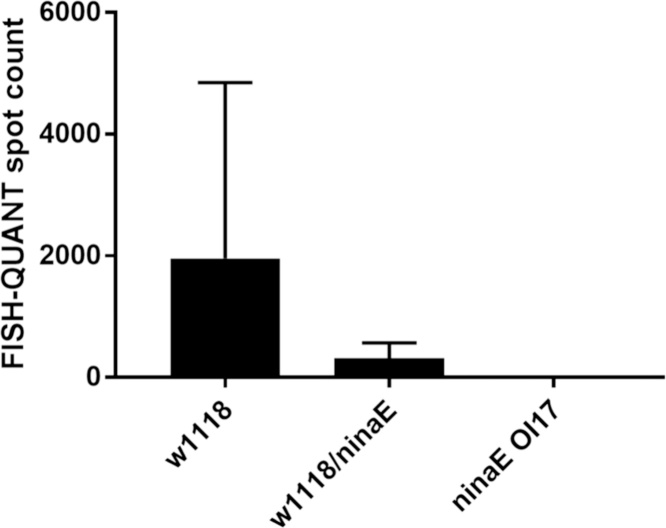
FISH-QUANT transcript counts for each *Drosophila* specimen genotype. FISH-QUANT utilizes settings chosen to recapitulate our conventional (LoG) spot counting algorithm. n = 3.

We further analyzed our in-house automated spot counting algorithm, comparing it against the widely used and verified open-source image processing software FISH-QUANT [8]. In order to compare the results of our conventional spot counting algorithm and to those of FISH-QUANT, we utilized identical FISH-QUANT parameter settings to those used in our automated spot counting algorithm. In particular, we used LoG filter settings hsize = 8 and sigma = 0.8. Starting with our simple-article genotype smFISH images, we performed FISH-QUANT pre-detection and selected an intensity threshold at the plateau region on the spot-intensity curve. We then selected a conservative spot “quality” score (see the FISH-QUANT user manual for a discussion of this metric) to threshold out all but the most likely transcript spots. Having used the simple-article genotype data to determine the intensity and quality thresholds, we proceeded to analyze the heterozygous and wild type smFISH data using the same threshold values and other FISH-QUANT settings. As expected, FISH-QUANT reliably identified spots in the expected cell regions (see representative images in Fig. S3) and spot counts agreed with expectations for mRNA transcript levels in WT (*w^1118^)*, heterozygous (*w^1118^/ninaE*^*0*117^*)*, and homozygous knockouts (*ninaE*^*0117*^ mutant) (Fig. 3).

We attributed the difference in output from our in-house automated spot counting algorithm and the FISH-QUANT analysis to our algorithm’s lack of a spot quality threshold parameter. Because FISH-QUANT features a quality thresholding parameter, the program accommodated possibly non-optimal choices for LoG filtering parameters. That is, because our spot counting algorithm’s output depended primarily on pre-selected LoG filtering parameters, even subtle adjustments to these parameters may result in dramatic shifts in reported spot counts. In an automated algorithm such as ours, systematic optimization of LoG filtering parameters is computationally limiting. Thus, we determined that our automated conventional spot counting methodology as-implemented does not consistently and accurately reflect levels of gene expression, at least in this complex *Drosophila* tissue specimen.

Our main goal was to develop an automated algorithm with which to analyze large image sets. Having identified that conventional LoG imaging analysis may not be the optimal method, we developed an alternative approach to conventional automated spot counting, discussed in the next section.

### Cell-by-Cell relative integrated transcript (CCRIT) quantification

**Fig. 4:**
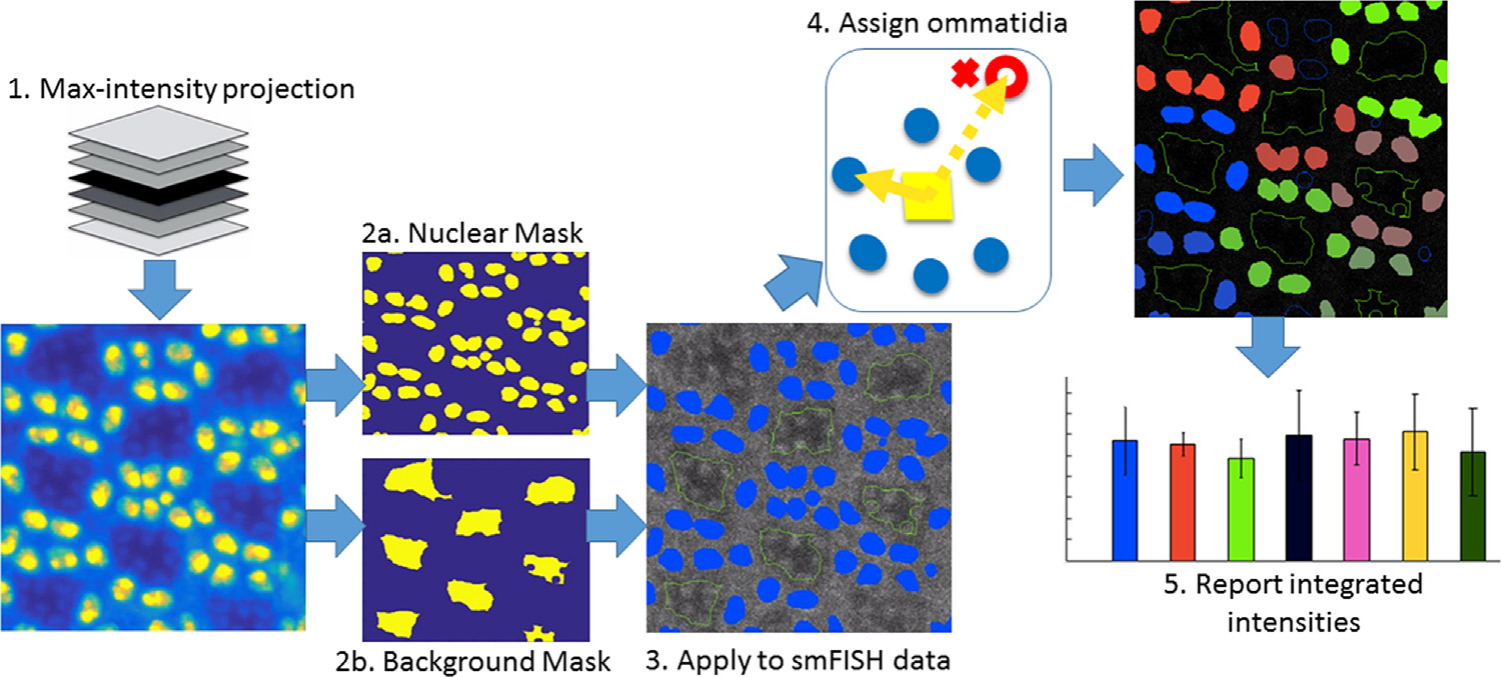
CCRIT uses a maximum intensity projection (1) to develop a nuclear and background mask (2) that identifies photoreceptor neuron clusters (3). After assigning photoreceptors to separate ommatidia (4), the algorithm averages the integrated smFISH signal intensities across all images in the multi-stack and reports the data for each ommatidium (5).

To identify individual photoreceptor neurons among distinct ommatidia and then quantify mRNA expression levels in each neuron, we have developed a set of algorithms that we designate Cell-by-Cell Relative Integrated Transcript (CCRIT) Quantification. CCRIT quantification is accomplished by the following process (summarized schematically in Fig. 4): (1) Maximum intensity projection, (2) Mask construction, (3) Gaussian filtering, (4) Photoreceptor identification and assignment, and (5) Intensity integration for each photoreceptor [9].

#### Masking

Because masking should be consistent across all images in a stack, our new algorithm first assigned a maximum intensity projection (MIP) from a z-stack of images in the DAPI (blue) channel. Each pixel in the MIP image received the maximum intensity observed for that pixel among the ensemble z-stack. The MIP is used to create two distinct masks: the (photoreceptor) nucleus mask and a background mask. The nucleus mask is used to identify individual photoreceptor neurons, and the background mask provides a digital noise subtraction.

We constructed the nucleus mask by first grouping the MIP’s pixel intensity range into 100 equally-sized bins. Each bin constituted a candidate masking threshold, chosen to be the maximum intensity value of that bin. For the nucleus mask, we found that selecting the central bin’s intensity threshold is consistently adequate. That central threshold was applied to the MIP: pixels above the threshold were assigned a value of 1, any below the threshold were assigned a value of 0. This binary nucleus mask was applied to each image in the stack, primarily to identify individual photoreceptor neurons.

We constructed the background mask by investigating the MIP for contiguous areas. First the MIP’s pixel intensity range was binned into 100 equally-sized bins to generate 100 candidate masks each with a masking threshold, again chosen to be the maximum intensity value of each bin. For each candidate mask a value of 1 was applied to all pixels in the MIP that were less than the candidate masking threshold value, and a value of 0 was applied to all pixels that were greater than the threshold value. For each candidate mask, thresholding resulted in many connected binary pixels (Binary Large Objects, or “blobs”). Blob area and “solidity” were readily calculated to assess each masking candidate. Solidity was defined as the ratio of a blob’s area to its convex area, which was determined using convex hulls—equivalent to stretching an elastic band around a blob’s boundary (in Matlab, refer to the convhull() command). The average blob solidity for each candidate mask was weighted proportionally to total blob area. Upon ranking each mask’s average weighted solidity as a function of candidate threshold intensity, we found that the smallest weighted solidity consistently corresponded to the most appropriate and robust background threshold intensity. The chosen background mask was then refined with a series of filters: opening, erosion, and infill filters, which all have distinct Matlab commands (imopen, imrode, and imfill). For each filter we used parameter values determined to be appropriate for the photoreceptor neuron, but that may need to be validated for other cell types. The opening filter removed small blobs (opening size = 50), defined with a structural element that also has a unique Matlab command (strel(‘disk’,5);). The erosion filter reduced overall blob size (erosion size = 10). Finally, the infill filter filled in holes within the remaining blobs (opening size = 1500). This final background mask is used to 1) assign photoreceptors to separate ommatidia and 2) inform a digital noise subtraction from each smFISH image.

#### Assigning cells

With masking completed, the resulting blobs (putative photoreceptor neurons) were automatically grouped into separate ommatidia based on the distances between each blob’s centroid. To that end, centroids are calculated for each blob from both the nucleus and background masks using Matlab’s centroid calculation function. Then the distances from each background blob centroid to every nucleus blob centroid was calculated. Using each background blob centroid as the reference, these distances were sorted into a distribution from least to greatest. A peak finder function was applied to the derivative of this distribution to identify an inflection point that distinguishes nucleus centroid distances that are close to a given background blob centroid and those that are distal. The nucleus blobs closest to each background blob were assigned to the same ommatidium. Photoreceptors within each ommatidium were numbered from 1 up to 7 arbitrarily in a counter-clockwise fashion about the ommatidium’s background blob.

#### mRNA transcript quantification

Noise subtraction on each smFISH image in the stack was performed to filter out signal that did not come from within the identified cells. First, the background mask was used as a sampling space in which an average intensity of pixels across the masked image was determined. This average “noise” intensity was subtracted from all pixels on each image in the multi-stack. Second, each digitally subtracted image was dot-multiplied with the nucleus mask, eliminating any extracellular signal. As a third and optional step, a 2D Gaussian filter smoothed the remaining smFISH signal. The filtered images were then ready for intensity quantification. mRNA transcript levels of each cell in each filtered image were quantified by calculating the integrated pixel intensity within each identified photoreceptor, and the data was organized by ommatidium. The data was reported in three ways: (a) averaged integrated intensity across all images in the stack, (b) integrated intensities averaged across the center 80% of images within the stack, and (c) from maximum intensities slice in across the stack.

### Validating the CCRIT method

**Fig. 5:**
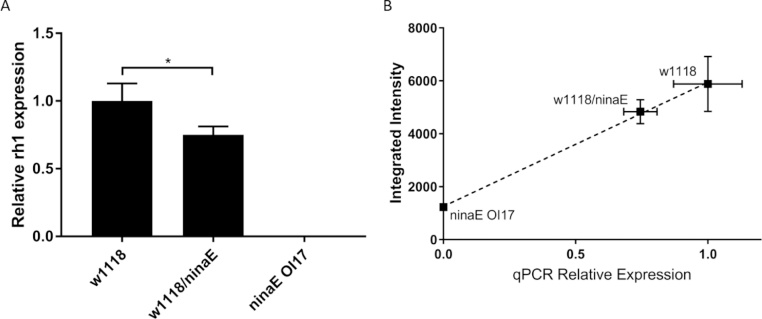
A) Relative transcript levels of Rh1 in wild-type and *ninaE* mutant adult heads. qRT-PCR analysis of Rh1 transcript levels in cDNA from wild-type (*w^1118^*) and *ninaE^o117^* homozygous and heterozygous adult flies. Mean transcript levels for each gene were normalized to *Rpl32* and plotted relative to the wild type, which was set to one. Error bars denote standard deviation for three biological replicates. B) Analysis of CCRIT against qPCR of *Drosophila* Rh1 mutants. qPCR data is normalized against the WT case. Error bars denote standard deviation from n = 5 specimens.

To validate that the CCRIT method provides reliable and quantitative data relative to standard measures we compared the CCRIT results against qRT-PCR data for each of three *Drosophila* genotypes (WT, heterozygous *ninaE*^*oI17*^, and homozygous *ninaE*^*o117*^). Quantitative reverse-transcription polymerase chain reactions (qRT-PCR) of wild-type and *ninaE* mutant adult heads was conducted with probes against Rh1 transcripts. We observed that approximately 70% of wild-type Rh1 transcript levels in heterozygous *ninaE*^*oI17*^ flies and no detectable Rh1 transcripts in the homozygous mutants (Fig. 5A).

We assessed Rh1 transcript expression by quantifying smFISH data for five specimens of each genotype (WT, heterozygous *ninaE^oI17^*, and homozygous *ninaE*^*o*I17^) using CCRIT. *Drosophila* photoreceptors are organized in radially symmetric clusters. As expected, six of the radial cells, R1–R6, were positive for the Rh1 smFISH probes. To facilitate CCRIT identification of individual cells, MIP-based masks were used to identify the positions of individual cells and assign them to ommatidia. The CCRIT GUI then non-discriminately labeled up to seven cells per identified ommatidium (note that R8 nuclei are not visible in the planes used for these analyses). Total smFISH pixel intensities were reported for each cell. We assume that the Rh1 negative cells (i.e. R7) do not contribute to the overall pixel intensity. For comparison against the qPCR data, the CCRIT integrated intensity values were averaged across all photoreceptors and all ommatidia identified in a stack of confocal microscopy data.

Plotting the integrated intensity values against the relative Rh1 expression level quantified by qRT-PCR (Fig. 5B) we found a strong linear correlation between both quantification methods (R^2^ = 0.993). Comparing the CCRIT results of the three genotypes, we found that the CCRIT integrated intensity values for the homozygous *ninaE^o117^* mutant were significantly different than the integrated intensity values for the heterozygous *ninaE^o117^* mutant (p-value < 0.01). CCRIT integrated intensity values for the WT and heterozygous mutant were also significantly different (p-value < 0.01). This is true despite considerable biological variability as well as our assumption that Rh1 negative cells do not contribute to the overall signal. Taken together, we conclude that CCRIT is an effective method for quantifying smFISH-labeled RNA expression in such crowded, noisy environments as the *Drosophila* photoreceptor.

### CCRIT graphical user interface

**Fig. 6:**
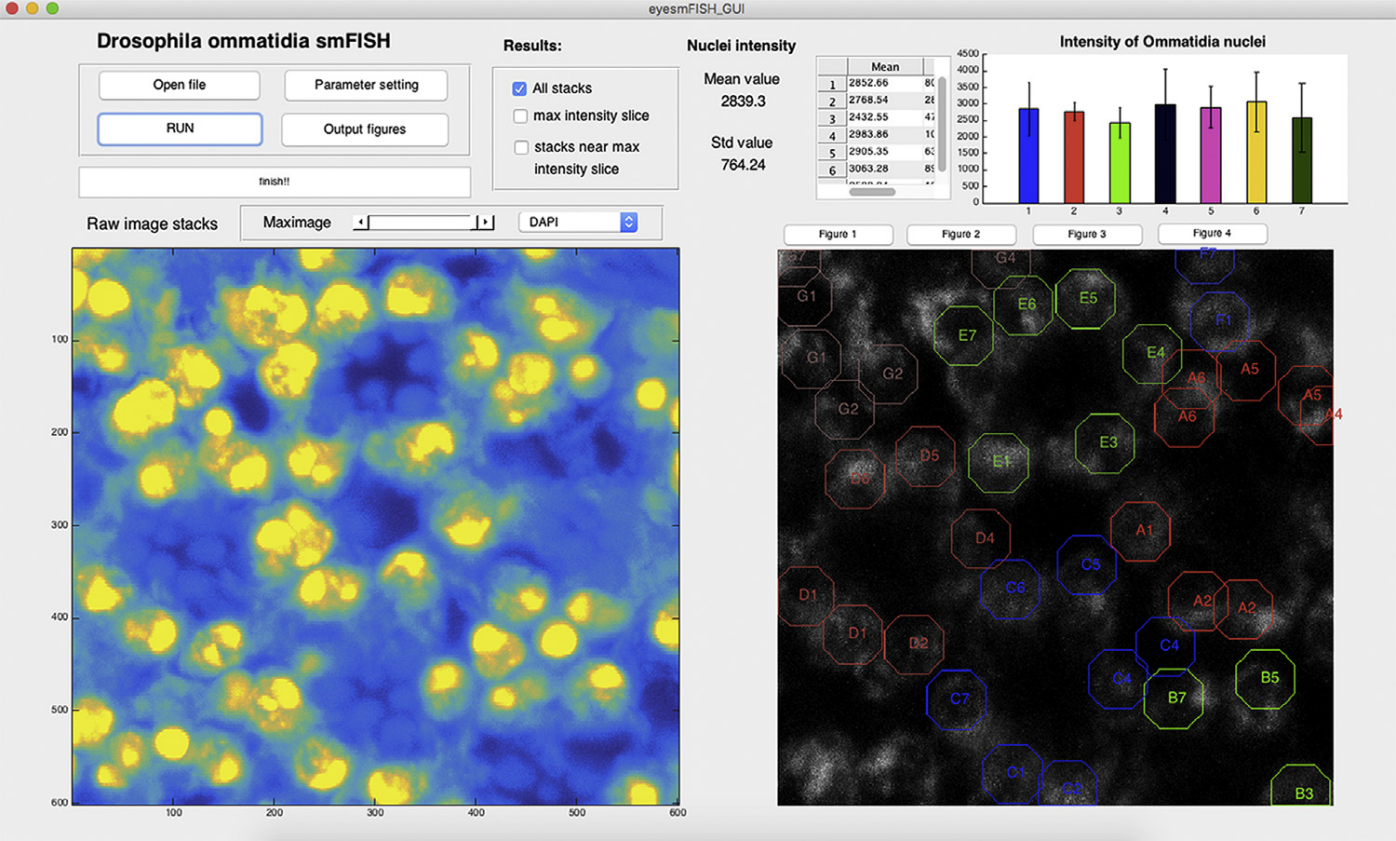
Representative display of our CCRIT user interface. At top left, raw microscopy data and user settings are entered. The desired result output data is also selected. At lower left a false-colored image of MIP, the DAPI-stained channel summed across all slices. Yellow denotes high intensity values and blue indicates the lowest intensity values. At lower right is a representative smFISH image with putative cells labeled by letters (denoting parent ommatidium) and numbers (denoting position within that ommatidium). At top right user selected output are graphically displayed (mean integrated intensity values with standard deviation).

To make the CCRIT method widely available to collaborators and the wider scientific community, we developed an easy to use graphical user interface (GUI) (Fig. 5) with which to implement the CCRIT method. The CCRIT GUI was designed to be applicable for a variety of data needs. At the top left of the interface window, the user can open the microscopy data file of interest, modify filtering parameters, select figures for output, and ultimately run the program. A false color image of a MIP for *Drosophila* eye photoreceptor neurons is shown in the left window, with yellow indicating pixels that have the strongest summed intensity values (nuclei stained with DAPI) and blue indicating the lowest intensity values. The right window shows an smFISH image where each photoreceptor centroid is marked by a colored octagon and labeled by a letter (denoting its parent ommatidium) and number (denoting its position with that ommatidium, ranging from 1 to 7 and assigned in an arbitrarily counter-clockwise fashion). We number cells from 1 to 7 because, as noted, ommatidium cell R8 does not appear in the analyzed microscopy planes. At the top right of the GUI window, descriptive statistics of each of the cells (averaged across all ommatidia and then through the entire multi-stack) are reported. The user may adjust the reported distributions to describe either “All slices”, the “max-intensity slice”, or “near the max intensity slice”, as set using the checkbox section (Fig. 6).

## Summary

Overall the workflow of the CCRIT method can be summarized as follows: 1) Create MIP, 2) Group MIP intensities into 100 groups, 3) Construct nucleus mask and identify photoreceptors, 4) Construct background mask and assign cells, 5) Apply masks to smFISH multi-stack, 6) Gaussian filter the remaining smFISH signal, 7) Report integrated smFISH signal intensity in each cell. Key distinctions of CCRIT to conventional spot counting techniques include: exclusion of Laplacian filtering, summation of the raw multi-stack for consistent image masking, ommatidium identification, and quantification by pixel intensity integration. Exclusion of a Laplacian filter accounts for data of uniformly-low pixel intensity, such as that for smFISH images of *Drosophila* that lack Rh1 transcripts (*ninaE^o117^*). Indeed, Laplacian filtering frequently exaggerates noise, perhaps accounting for the unexpected result shown in Fig. 2F. In future editions, our CCRIT algorithm will intelligently identify the *Rh1*-negative photoreceptors for use as internal baselines of RNA expression within each ommatidium (prohibitively, the position of the *Rh1*-negative cell is different for each of the many ommatidia visible in a given image slice because ommatidia in each half of the compound eye mirror each other).

We find that our in-house automated spot counting algorithm, though employing conventional methods, was not appropriate for analyzing large microscopy image data sets. Parameter optimization was computationally restrictive, especially for images containing crowded or signal or having moderate signal-noise ratios. Widely available spot counting algorithms such as FISH-QUANT, while robust and reliable, require significant user input and are not ammenable to automated high-throughput analysis of large data sets. CCRIT overcomes these problems by automatically identifying regions of interest in an image (i.e. cells) and integrating pixel intensities. Moreover, CCRIT accommodates noise by forgoing Laplacian filtering. We have shown that CCRIT can be effectively applied to large confocal microscopy data sets to accurately assess RNA transcript expression levels of Rh1 transcripts in a set of *Drosophila* mutants. Our GUI implementation of CCRIT constitutes a powerful and translatable tool for studying single-cell transcriptomes. We expect CCRIT to be readily translatable to single cell mRNA transcript quantification in a variety of contexts including complex cellular geometries and large confocal image sets across various model systems.
