# Supplementary material for: An automated workflow for quantifying RNA transcripts in individual cells in large data-sets
Source: MethodsX. 2017 Sep 1;4:279–88. doi: 10.1016/j.mex.2017.08.002 (PMC5596354; doi:10.1016/j.mex.2017.08.002)
Supplement: Supplementary file 1 [file mmc1.pdf]

Supplementary Material:

Sample data and conventional spot counting LoG algorithms. Pharris, M. C.; Wu, T.; Chen, X.; Wang, X.; Umulis, D.; Weake, V. M.; Kinzer-ursem, T. L. (2017), "Pharris et al. MethodsX (2017) Supplemental Data Set." (DOI: 10.4231/R7M043DC).

smFISH of adult *Drosophila* eyes

The smFISH protocol for adult *Drosophila* eyes was modified from the protocol described for smFISH of *Drosophila* oocytes and embryos[1]. Adult male flies aged one day post-eclosion were anaesthetized with CO_2, decapitated, and the heads were transferred to cold fresh fixation buffer (4% paraformaldehyde, 1x PBS, 0.1% DEPC) on an agar dissecting plate. Eyes were dissected in fixation buffer using fine dissection scissors (Fine Science), trimmed to remove excess cuticle and hairs, then scooped using a curving straight needle and transferred to new fixation buffer in a 1.5 mL Eppendorf tube on ice for 0.5h. All reagents and consumables post-fixation were prepared using RNAse-free materials. Following fixation, eye tissue was dehydrated as follows: eyes were washed three times with PBST (1x PBS, 0.1% Tween-20, 0.1% DEPC) for 5 min each time, then sequentially incubated in 30% methanol (in PBST), 50% methanol, 70% and 100% methanol for 5 min each time. All washes were performed in 1.5 mL Eppendorf tubes using ice-cold buffers. Following dehydration, eye tissue was rehydrated as follows: eyes were incubated sequentially in the following solutions for 15 min each time: 70%, 50%, 30% methanol (in PBST), and finally PBST only. Next, eyes were incubated in washing buffer (4x SSC, 35% deionized formamide, 0.1% Tween-20, 20 mM 2-mercaptoethanol) for 30 min, transferred to Hybridization buffer with probes (0.5 nM probes in 50 μL hybridization buffer) for 5 h in the dark at 37°C (using a water bath). Eyes were then washed twice with pre-warmed washing buffer for 30 min each time. Incubation buffer supplemented with probes was stored at -20°C for short-term use or -80°C for long-term storage. Eyes were stained with 4',6-diamidino-2-phenylindole (DAPI) at a final concentration of 2.5 ng/mL in PBST. Eyes were mounted in H-1000 vectashield medium (Vector Laboratories, Inc, CA) on slides with coverslip attached with wax bridges on each corner, and imaged with LSM710 (Zeiss) confocal microscope using a 63x lens with 4X zoom. For each eye, z-stack images of 0.65 μm slices for 20 μm total depth were collected at pixel resolutions of 512x512 or 1024x1024.

qPCR analysis of gene expression

RNA was extracted from heads from five male flies aged one day post-eclosion. 100 ng of DNAse-treated RNA was used to generate cDNA and transcript levels of *rh1* and *Rpl32* were determined using qPCR as previously described relative to a dilution series of cDNA standards[2]. Three biological replicates were examined for all genotypes. The following primers were used for qPCR analysis: *Rpl32* 5' GCTAAGCTGTCGCACAAATG 3' and 5' CGTTGTGCACCAGGAACTT 3'; *rh1* 5' GGAGCAGGTATGTGCCGGAGGGTAAC 3' and 5' TGGGCGGAGACAGCAGCAATGATGAAC 3'.

Calculation of point-spread function for microscope used in *Drosophila* imaging.

We imaged samples with 100nm beads made from Zeiss. PSF is related to the wavelength of light, pinhole, NA of objective and the size of objective. We used the same parameters when imaging smFISH signals. We used the 647 laser with 1.38 Airy unit pinhole by 63* oil objective (NA 1.4).

During imaging, we sampled the lateral xy at 84nm and axial z at 200 nm, using one directional scanning and 4 times zoom. We collected 34 individual microspheres shown in Figure S1 which are labeled with white circles.

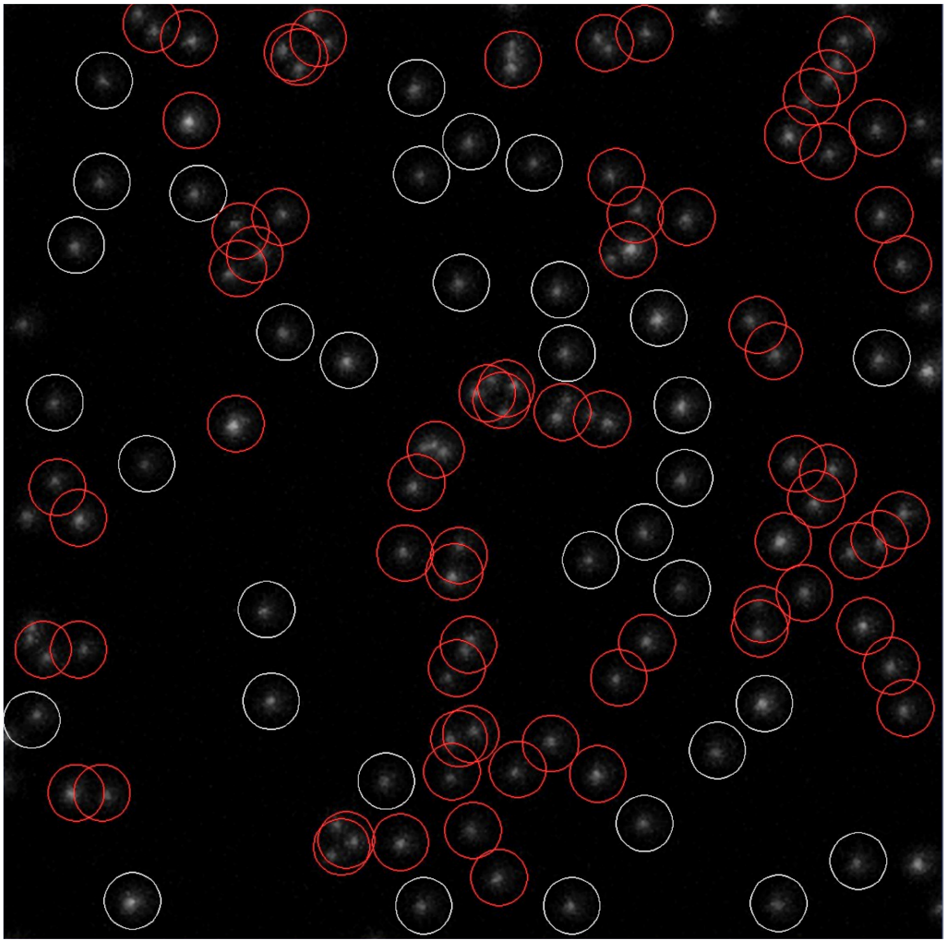

Figure S1. Beads selected to calculate PSF are labeled with white circles; red circles mark the beads rejected due to unacceptable shape or position relative to other beads.

Then, we averaged 34 samples to one SPF which are shown in Figure S2.

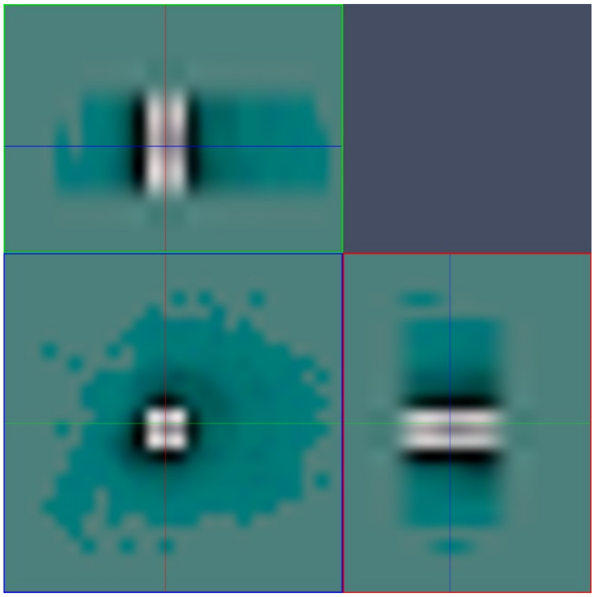

Figure S2. Representative PSF orthogonal views showing xy image (lower left), the xz image plane (top) and yz image plane (right).

From figure 2, xy,xz and yz are fitted to a Gaussian function. And we used the figure to calculate the full-width at half-maximum (FWHM).

The final output are shown as below:

FWHM(X)=378nm;

FWHM(Y)=387nm;

FWHM(Z)=475nm.

We used 100nm beads and sample size 34.

Also, based on the imaging settings, we calculate the Nyquist limit for xy=252nm, and the Nyquist limit for z=400nm.

<u>**Visualization of Representative FISH-QUANT Results**</u>

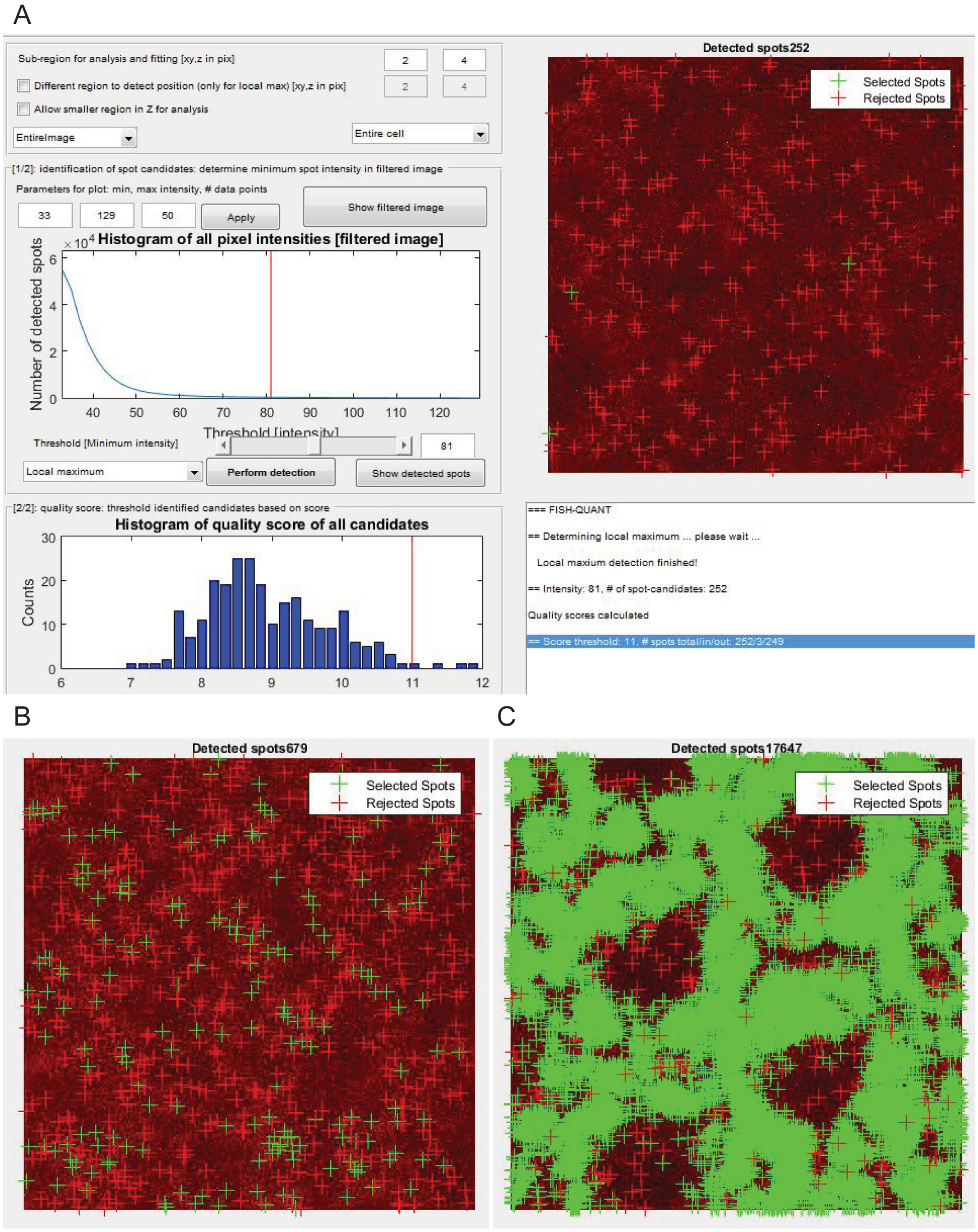

Figure S3. Representative FISH-QUANT screenshots visualizing spot detection in each *Drosophila* genotype (data introduced in Figure 2), using settings chosen to recapitulate our CCRIT algorithm. A) Pre-detection of the null genotype, which was used in determining intensity and spot quality thresholds

for counting in the heterozygous (B) and wild type (C) images. Putative spots rejected in FISH-QUANT are shown in red; accepted spots are shown in green.

Supplemental References:

[1] E. Abbaszadeh and E. Gavis, "Fixed and live visualization of RNAs in Drosophila oocytes and embryos", Methods, vol. 98, pp. 34-41, 2016.

[2] J. Ma and V. Weake, "Affinity-based Isolation of Tagged Nuclei from Drosophila Tissues for Gene Expression Analysis", Journal of Visualized Experiments, vol. 85, 2016.

[3] J. O'Tousa, W. Baehr, R. Martin, J. Hirsh, W. Pak and M. Applebury, "The Drosophila ninaE gene encodes an opsin", Cell, vol. 40, no. 4, pp. 839-850, 1985.
